# Optimizing forage harvest and the nutritive value of Italian ryegrass-based mixed forage cropping under northwestern Himalayan conditions

**DOI:** 10.3389/fpls.2024.1346936

**Published:** 2024-07-03

**Authors:** Naveen Kumar, Rajender Singh, Rajiv Kumar Agrawal, Gurbhan Dass Sharma, Akashdeep Singh, Tarun Sharma, Ranbir Singh Rana

**Affiliations:** ^1^ Department of Agronomy, Chaudhary Sarwan Kumar Himachal Pradesh Krishi Vishvavidyalaya, Palampur, India; ^2^ Project Coordination Unit, All India Coordinated Research Project on Forage Crops, ICAR–Indian Grassland and Fodder Research Institute, Jhansi, Uttar Pradesh, India; ^3^ National Agricultural Higher Education Project (NAHEP) - Centre of Advanced Agricultural Science & Technology (CAAST), Chaudhary Sarwan Kumar Himachal Pradesh Krishi Vishvavidyalaya, Palampur, India

**Keywords:** Egyptian clover, forage harvest, genotypes, Italian ryegrass, mixed cropping, nutritive value, seeding ratios

## Abstract

The scarcity of high-quality forage has a significant influence on the productivity and profitability of livestock. Addressing this concern, an investigation was undertaken to assess the effects of distinct Italian ryegrass genotypes, namely, Punjab ryegrass-1, Kashmir collection, and *Makhan* grass, in conjunction with varying seeding ratios of Italian ryegrass to Egyptian clover. The seeding ratios considered were 100:0 (Italian ryegrass to Egyptian clover), 75:25, 50:50, and 25:75. All possible combinations of Italian ryegrass and Egyptian clover with seeding ratios were set up in a randomized complete block design and replicated thrice. Co-cultivating Italian ryegrass and Egyptian clover at a 75:25 seeding ratio yields the best yield benefit, as determined by the land equivalent ratio. It is noteworthy that in this configuration, real yield loss is higher for Egyptian clover and for Italian ryegrass when the seeding ratio is 25:75. The higher competitiveness of Italian ryegrass in comparison to Egyptian clover is highlighted by the competitive ratio. Notably, the nutritive parameter, crude protein yield, was significantly higher in the *Makhan* grass-based 50:50 and 75:25 seeding ratio. Results of the study ascertained the compatibility of grass-legume co-cultivation with significantly higher quantity and quality forage harvested under mixed cropping systems whereas *Makhan* grass as the superior and dominant genotype in comparison to Kashmir collection. The outcomes of this study revealed that the 100:0 seeding ratio, coupled with the *Makhan* grass genotype, exhibited superior performance in terms of cumulative forage harvest, dry matter accumulation, net returns, and benefit–cost ratio.

## 1 Introduction

Italian ryegrass (*Lolium multiflorum* Lam.), a winter forage grass of Southern Europe, is cultivated throughout Australia, North America, Europe, and New Zealand as a short-lived grass for its great digestibility and palatability (Lithourgidis et al., 2006). Being farmed across the temperate zones of the world, Italian ryegrass is a very productive and nutritive grass that is capable of soil conservation and is particularly excellent for waterlogged soils (Hannaway et al., 1999; Gerdes et al., 2005; Meena et al., 2017; Salama, 2020). Wider adaptability and capability to produce greater quantities of quality forage biomass makes Italian ryegrass a better candidate for mixed cropping systems along with legumes (Thalooth et al., 2015). Several desirable characteristics such as quick establishment, suitability for reduced tillage, and nutritional multicuts make Italian ryegrass a preferable winter forage crop (Meena et al., 2017).

Egyptian clover (*Trifolium alexandrinum*), a native of Western Asia, is an annual leguminous forage crop frequently planted in the southern United States and nations with moderate winters, such as India, Egypt, Turkey, and Pakistan (Pecetti et al., 2012). With its restorative character and vast adaptability across diverse soil types, including soils with salinity and alkalinity concerns, its cultivation is being encouraged to such soils where commercial crop cultivation meets a slew of challenges. Cattle breeding and milk production are inextricably tied with forage production. Winter is the most restricted season, with less green forage available for the animals. Egyptian clover is used for both seed and forage, as well as a multifunctional forage crop in the winter, producing green forage, hay, and silage (Tufail et al., 2020; Abuzaid et al., 2021). Because of its great nutritional content, the crop is a key component of the animal diet. It has a high concentration of crude protein (15.8%–26.7%), crude fiber (14.9%–28.3%), calcium (1.4%–2.58%), nitrogen (2.52%–4.25%), and phosphorus (0.14%–0.20%) (Devi, 2019). Egyptian clover is a popular forage crop among farmers worldwide due to its multicut nature, great palatability, ability to fix atmospheric nitrogen, and rapid development (Tufail et al., 2020; Jabbar et al., 2022). In the face of climate change, the crop has high water requirements as well as susceptibility to temperature changes (Salama, 2020).

In addition to increasing animal production, mixed cropping is a great way to address the wintertime forage deficit (Meena et al., 2017). It is among the effective strategies that may generate high-quality food while using less resources sustainably. Legumes and grasses in cropping combinations provide forage mixtures with increased protein and fiber concentration, improving their nutritional value (Ansar et al., 2010). Aside from improving nutritive value, feed mixes are said to increase fodder consumption (Ansar et al., 2010). Combining forage legumes with annual grasses creates an optimal cropping system with characteristics such as complementary growth habit, high biomass, high protein, fiber, improved rhizosphere-based microbial activity, and minimal input demand (Rakeih et al., 2008; Salama, 2020). Forage-legume-based mixed cropping systems offer high production and resource usage efficiency due to their diverse ability to utilize resources efficiently such as light, water, and nutrients (Lithourgidis et al., 2006; Atis et al., 2012). Such cropping systems are far less vulnerable to insect and plant invasions, as well as weather changes that are common in monocultures (Lithourgidis et al., 2011). Yucel et al. (2018) proposed Italian (annual) Ryegrass–Egyptian clover mixed cropping systems as a potential choice for excellent forage production with substantially higher quality forage production with 60% Egyptian clover and 40% annual ryegrass-based mixture. Magda et al. (2021) studied the Egyptian clover–annual ryegrass-based intercropping systems along with the influence of nitrogen fertilizers on such systems and found that a 75:25-based sowing proportion of annual ryegrass and Egyptian clover was the best in terms of forage harvest and nutritive value. Rady et al. (2022) studied the Egyptian clover-based monoculture systems as well as intercropping systems with grasses including triticale, ryegrass, barley, and oats and observed better forage harvests and nutritive value for grass–legume-based intercropping systems.

Mixing ratios are a key determinant of the nutritive value and harvest that may be obtained from forage-legume-based mixed cropping systems due to the competitive nature of grasses compared to legumes. Italian ryegrass genotypes differ in their growth behavior and dominance over component crops, which might have a substantial impact on Egyptian clover’s growth capacity. Keeping in consideration the variable response of Egyptian clover and annual ryegrass at variable sowing proportions and differential growth ability of Italian ryegrass genotypes and their dominance over component crops, the present long-term field experiment was designed with an aim to evaluate and recommend Italian ryegrass genotypes and corresponding seeding ratios for enhanced forage harvest and optimized nutritive value in cropping mixtures with Egyptian clover. The objectives of the experiment were to ascertain forage harvests, assess their nutritive value, analyze competition involved among crops, and evaluate the economic benefits and soil fertility implications.

## 2 Materials and methods

A 4-year field experiment was conducted at Fodder Farm, Department of Genetics and Plant Breeding of Chaudhary Sarwan Kumar Himachal Pradesh Krishi Vishvavidyalaya, Palampur, India during the winter season from 2014 to 2018. The experiment began on 17 October 2014 and ended on 19 April 2018.

The Palampur region of Himachal Pradesh (HP), India, falls under the sub-temperate humid zone of HP, which is characterized by mild summers and cool winters. The experimental site lies between 32°6´N and 76°3´E at an elevation of 1,200 m AMSL (Above mean sea level). The soil at the experimental site was acidic in reaction (pH = 5.4), medium in organic carbon (0.68%), low in available nitrogen (217 kg/ha), and medium in available phosphorus (12 kg/ha) and available potassium (236 kg/ha).

Randomized complete block design (RCBD) was applied to the experiment wherein the experimental field was divided into three blocks that served as replicates. Each block was divided into 13 treatments wherein all possible combinations of three genotypes of Italian ryegrass and the four seeding ratios with the Egyptian clover along with the sole stand of Egyptian Clover were randomly allocated using random number table. The treatments consisted of Italian ryegrass genotypes, *viz.*, Punjab ryegrass-1, Kashmir collection, and *Makhan* grass and four seeding ratios for Italian ryegrass: Egyptian clover (100:0, 75:25, 50:50, and 25:75) and the sole stand of broadcasted Egyptian clover. *Makhan* grass was being tested for release in the state of Himachal Pradesh, whereas the Kashmir collection and Punjab ryegrass-1 were the standard checks for the *Makhan grass* genotype. The Makhan grass genotype has been known to have a plant height ranging from 10 to 40 cm along with rapid-growing, high forage-producing capacity and crude protein concentration highly suitable for most of the livestock classes (Tiwari et al., 2023). Punjab ryegrass-1, on the other hand, a multicut genotype of annual ryegrass, had an average forage harvest yield of 32.5 Mg/ha along with the presence of soft stems and leaves in its biomass (Kushwaha and Singh, 2023). The Egyptian clover variety used was Mescavi.

Traditional tillage procedures were practiced before sowing the crop seeds as per the experimental treatments. Italian ryegrass was sown at a rate of 15 kg/ha, whereas Egyptian clover was sown at a rate of 25 kg/ha. Application of nutrients, such as nitrogen:phosphorus:potassium, using urea (46% nitrogen), single super phosphate (16% phosphorus), and muriate of potash (60% potassium), was applied at a rate of 120:60:40 kg/ha when Italian ryegrass was grown as the sole or component crop, and at a rate of 20:60:40 kg/ha when Egyptian clover was used as the sole crop. At the time of sowing half of the recommended nitrogen, full dose of phosphorus and potassium were applied as basal dose in both sole ryegrass and berseem cropping as well as in their intercropping systems. The remaining nitrogen was split into four portions each applied 30 days after sowing (DAS) and at first cut, second cut, and third cut, respectively. Irrigation and weed management practices were maintained uniformly for all the plots. For irrigation (pre-sowing irrigation + four irrigations per season) and weed management (single time), the surface method and the hand weeding method were practiced every season.

### 2.1 Sampling and measurements

#### 2.1.1 Total forage harvest and dry matter accumulation

Four cuttings, the first at 78 DAS, the second at 108 DAS, the third at 143 DAS, and the fourth at 183 DAS, were sampled from the different treatments. With a field stubble of 10 cm, the plants were trimmed using stainless steel sickles. Biomass from the entire plot was gathered for green forage, discarding the plot’s border rows. The total forage harvest was derived with the summation of forage harvest obtained for each cut. Single forage harvest value for each cut was derived by averaging the total forage harvest obtained each cut each year with number of years. The dry matter accumulated was determined by taking a 100-g sample of fresh green forage from the net plot area, air-drying it for 3–4 days, and then oven-drying it at 105°C in a hot air oven to achieve a consistent weight. Total dry matter accumulation was derived using the same procedure as green forage harvest.

#### 2.1.2 Crude protein concentration and crude protein yield

The samples were further ground in a stainless-steel-based grinder to a particle size of 1 mm for nutritive analysis. The nutrient concentration of plant samples was determined using standard procedure for nitrogen concentration (Jackson, 1973). The crude protein content was obtained by multiplying nitrogen concentration with a factor of 6.25. Subsequently, crude protein yield was obtained by using the following formula:


$$Crude\;protein\;yield=\frac{Crude\;protein\;concentration\times dry\;matter\;accumulated}{100}$$

#### 2.1.3 Soil analysis

The soil samples were collected at the end of each season with auger from 0- to 15-cm soil depth and taken to the laboratory for drying, and for further processing and analysis. The soil organic carbon and available nitrogen were determined using standard procedures for organic carbon, available nitrogen, potassium, and phosphorus, i.e., wet digestion method (Walkley and Black, 1934), alkaline permanganate method (Subbiah and Asija, 1956), neutral normal ammonium acetate extraction method (Black, 1965), and Olsen’s method (Olsen et al., 1954), respectively.

#### 2.1.4 Competitive indices

##### 2.1.4.1 Land equivalent ratio

Land equivalent ratio (LER) defines the efficiency of cropping mixtures in utilizing environmental resources efficiently (Mead and Willey, 1980). LER can be derived as given below (Rady, 2016):


LER=LER (Egyptian Clover) x LER (Italian Ryegrass)

$$LER\;\left(Egyptian\;Clover\right)=\frac{Y\left(EC_{IR}\right)}{Y\left(EC\right)}$$

$$LER\;\left(Italian\;Ryegrass\right)=\frac{Y\left(IR_{EC}\right)}{Y\left(IR\right)}$$

where Y (EC_IR_) = yield of Egyptian clover as an intercrop in Italian ryegrass, Y (IR_EC_) = yield of Italian ryegrass as an intercrop in Egyptian clover, Y (EC) = yield of Egyptian clover as a sole crop, and Y (IR) = yield of Italian ryegrass as a sole crop.

A value of 1 is critical in assessing LER; when it is greater than 1, then the cropping mixture favors the growth and productivity of mixing partners, and when it is less than 1, there is a negative impact over growth and productivity of mixing partners in that cropping mixture.

##### 2.1.4.2 Competitive ratio

Competitive ratio (CR) describes the competition occurring between crop species involved in a particular cropping mixture (Willey and Rao, 1980). It defines the competitive ability of involved crop species and is an advantageous alternative to aggressivity. CR can be derived based on the equation below (Rady, 2016):


$$CR\;\left(Egyptian\;CLover\right)=\frac{LER\left(Egyptian\;CLover\right)}{LER\left(Italian\;Ryegrass\right)}x\frac{Z\left(IR_{EC}\right)}{Z\left(EC_{IR}\right)}$$

$$CR\;\left(Italian\;Ryegrass\right)=\frac{LER\left(Italian\;Ryegrass\right)}{LER\left(Egyptian\;Clover\right)}x\frac{Z\left(EC_{IR}\right)}{Z\left(IR_{EC}\right)}$$

##### 2.1.4.3 Relative crowding coefficient

Relative crowding coefficient (*K*) is basically an index to measure the relative dominance of one crop species over another in a cropping mixture (De Wit, 1960). It can be calculated using the equation given below (Rady, 2016):


K (combined)=K (Egyptian Clover) x K (Italian Ryegrass)

$$K\;\left(Egyptian\;Clover\right)=\frac{Y\left(EC_{IR}\right)\;x\;Z\left(IR_{EC}\right)}{Y\left(EC\right)-Y\left(EC_{IR}\right)}x\;Z\left(EC_{IR}\right)$$

$$K\;\left(Italian\;Ryegrass\right)=\frac{Y\left(IR_{EC}\right)\;x\;Z\left(EC_{IR}\right)}{Y\left(IR\right)-Y\left(IR_{EC}\right)}x\;Z\left(IR_{EC}\right)$$

where Z (IR_EC_) = seeding proportion of Italian ryegrass in mixture and Z (EC_IR_) = seeding proportion of Egyptian clover in mixture.

Interpretation for relative crowding coefficient values can be assigned as follows:

When the value of [*K* (Egyptian clover) * *K* (Italian ryegrass)] is greater than 1, then there is a yield advantage; when it is less than 1, there is a disadvantage; and when it is equal to 1, there is a disadvantage or advantage.

##### 2.1.4.4 Actual yield loss

Actual yield loss (AYL) has been known to be a precise and efficient index to define the competition between and within component crops more accurately. It describes the behavior of the component crops in a particular cropping mixture as it takes into consideration the actual sown proportion and yield as pure and intercrops for participating crops. AYL can be derived based on the equation below (Banik, 1996):


AYL (combined)=AYL (EC)+AYL (IR)

$$AYL\left(EC\right)=\frac{\frac{Y\left(EC_{IR}\right)}{Z\left(EC_{IR}\right)}}{\frac{Y\left(EC\right)}{Z\left(EC\right)}}-1$$

$$AYL\left(IR\right)=\frac{\frac{Y\left(IR_{EC}\right)}{Z\left(IR_{EC}\right)}}{\frac{Y\left(IR\right)}{Z\left(IR\right)}}-1$$

Values of AYL can be positive or negative, signifying yield advantage or disadvantage for component crops.

### 2.2 Economic benefits

Net returns were derived by subtracting gross returns from the total cost of cultivation. The equation for which is given below:


Net returns=Gross returns−cost of cultivation

Benefit–cost ratio was derived by dividing gross returns by cost of cultivation for which the equation is given below:


$$Benefit–cost\;ratio=\frac{Gross\;Returns}{Cost\;of\;cultivation}$$

### 2.3 Data analysis

The dataset derived from both field and laboratory analyses were scrutinized by the analysis of variance technique within the framework of an RCBD. The 4-year data were subjected to homogeneity test of variance using Bartlett’s Chi-square test as prescribed by Gomez and Gomez in 1984. The Chi-square test was non-significant; therefore, the data were pooled over years without any transformation. The R packages “ggplot2” (Wickham, 2016), “agricolae” (Felipe, De, 2009), “dplyr” (Wickham et al., 2023), and “gridextra” (Auguie and Antonov, 2017) were utilized in data analysis and visualization, ensuring a comprehensive understanding of the obtained results. Significant treatment variations were assessed with a stringent threshold of a 5% level of significance (*p* = 0.05), adhering to established statistical protocols. Additionally, a correlation heat map was created, to study the correlation between the various yield parameters and competitive indices, using R-software. The R packages “ggplot2” (Wickham, 2016) for visualization of correlation heatmap and “GGally” (Schloerke et al., 2024) for calculating correlations were used (R Core Team, 2023).

## 3 Results

### 3.1 Green forage and dry matter accumulation harvested

Green forage harvested at various cuts was significantly influenced under the influence of annual ryegrass genotypes and the seeding ratios of Egyptian clover and annual ryegrass genotypes (**Table 1**). The initial harvest yield exhibited a marked increase in treatments employing Punjab ryegrass-1 at a seeding ratio of 75:25, followed closely by those utilizing a 50:50 seeding ratio of the same variety. The latter treatment demonstrated statistical parity with those incorporating the *Makhan* grass genotype at seeding ratios of 75:25, 50:50, and 100:0, as well as with the Kashmir collection at 100:0 seeding ratio. During the second cut, a significantly higher forage harvest was recorded with Punjab ryegrass-1 (50:50) followed by Punjab ryegrass-1 (75:25) and *Makhan* grass with seeding ratios of 50:50, and 75:25. A significantly higher forage harvest in third cut was recorded in treatments using *Makhan* grass at a seeding ratio of 100:0, followed by those with a seeding ratio of 75:25, and 50:50 of the same genotype and also with Punjab ryegrass-1 with seeding ratios of 100:0, 75:25, and 50:50. A comparable trend was recorded during the final cut of Italian ryegrass genotypes in combination with Egyptian clover at varied seeding ratios. During the different cuts, significantly lower forage harvest was recorded where the sole stand of Egyptian clover was cultivated.

**Table 1:** Effect of seeding ratios and Italian ryegrass genotypes on cut-wise forage harvest.

Treatment	Cut-1	Cut-2	Cut-3	Cut-4
**T_1_: Punjab ryegrass-1 + 100:0**	12.57^bcd^	13.81^de^	16.55^b^	16.76^b^
**T_2_: Punjab ryegrass-1 + 75:25**	14.1^a^	15.8^bc^	16.4^b^	15.0^c^
**T_3_: Punjab ryegrass-1 + 50:50**	13.0^b^	17.0^a^	16.3^b^	10.8^e^
**T_4_: Punjab ryegrass-1 + 25:75**	12.2^bcd^	13.5^e^	12.7^d^	11.3^e^
**T_5_: Kashmir Collection + 100:0**	12.7^bc^	13.3^e^	13.9^cd^	10.1^ef^
**T_6_: Kashmir Collection + 75:25**	12.0^cde^	13.7^de^	13.2^cd^	6.6^g^
**T_7_: Kashmir Collection + 50:50**	11.7^de^	13.7^de^	12.5^d^	4.5^h^
**T_8_: Kashmir Collection + 25:75**	10.6^f^	12.7^e^	10.6^e^	7.5^g^
**T_9_: *Makhan* Grass + 100:0**	12.3^bcd^	14.8^cd^	19.3^a^	19.2^a^
**T_10_: *Makhan* Grass + 75:25**	12.6^bcd^	15.8^bc^	17.7^b^	16.6^b^
**T_11_: *Makhan* Grass + 50:50**	12.6^bcd^	16.2^ab^	16.9^b^	16.1^bc^
**T_12_: *Makhan* Grass + 25:75**	11.3^ef^	13.6^e^	14.5^c^	12.6^d^
**T_13_: Sole Egyptian clover**	9.8^g^	11.6^f^	10.7^e^	8.9^f^

Treatment means with different lowercase letters are significant different as determined by Duncan’s Multiple Range Test (DMRT).

Similarly, the total amount of forage harvested and dry matter accumulation were strongly influenced by the combination of Egyptian clover and several genotypes of Italian ryegrass under varying seeding ratios. The total amount of forage harvested and dry matter accumulated ranged from 40.96 to 65.61 Mg/ha and 7.24 to 13.95 Mg/ha, respectively. The sole stand of Italian ryegrass genotype, *Makhan* grass was proved to be the most productive with significantly higher dry matter and forage harvest produced i.e., 13.95 and 65.61 Mg/ha. *Makhan* grass did perform the best across the experimental years (2014–2018) with significantly higher green forage harvests and dry matter accumulation as compared to other sole stands as well as forage mixtures (**Tables 2**, **3**). Among forage mixtures, co-cultivating Egyptian clover and *Makhan* grass at seeding ratios of 75:25 produced significant equivalent results to cultivation of *Makhan* grass alone. Similar stable and consistent results with a forage mixture of Egyptian clover and *Makhan* grass at seeding ratios of 75:25 especially in terms of green forage harvest were observed across the experimental study from 2014 to 2018 (**Table 3**). Punjab ryegrass-1 did perform good with at par dry matter accumulation for the sole stand of Punjab ryegrass-1 (12.76 Mg/ha), whereas in terms of total forage harvest, it performed second best when sown at a ratio of 75:25 (61.23 Mg/ha) with Egyptian clover.

**Table 2:** Effect of seeding ratios and Italian ryegrass genotypes on forage harvest from 2014 to 2018.

Treatment	2014–2015	2015–2016	2016–2017	2017–2018
**T_1_: Punjab ryegrass-1 + 100:0**	58.37^bc^	58.84^bc^	60.04^bc^	61.53^cd^
**T_2_: Punjab ryegrass-1 + 75:25**	59.78^b^	60.51^b^	61.38^b^	63.28^bc^
**T_3_: Punjab ryegrass-1 + 50:50**	55.71^c^	56.67^c^	57.31^c^	59.04^d^
**T_4_: Punjab ryegrass-1 + 25:75**	48.28^d^	49.48^de^	49.88^d^	51.15^e^
**T_5_: Kashmir Collection + 100:0**	48.22^d^	48.58^e^	50.05^d^	53.04^e^
**T_6_: Kashmir Collection + 75:25**	43.97^e^	44.67^f^	45.57^e^	47.69^f^
**T_7_: Kashmir Collection + 50:50**	40.95^f^	41.78^g^	42.48^f^	44.35^g^
**T_8_: Kashmir Collection + 25:75**	39.67^f^	40.70^g^	41.27^f^	44.22^g^
**T_9_: *Makhan* Grass + 100:0**	64.08^a^	64.41^a^	65.85^a^	68.11^a^
**T_10_: *Makhan* Grass + 75:25**	61.26^ab^	61.90^ab^	62.66^b^	65.06^ab^
**T_11_: *Makhan* Grass + 50:50**	60.36^b^	61.30^b^	62.03^b^	63.40^bc^
**T_12_: *Makhan* Grass + 25:75**	50.55^d^	51.88^d^	52.08^d^	53.08^e^
**T_13_: Sole Egyptian clover**	39.52^f^	40.35^g^	41.19^f^	42.80^g^
**SEM±**	0.788	0.769	0.766	0.866
**LSD (*p<* 0.05)**	2.315	2.258	2.250	2.543

Treatment means with different lowercase letters are significant different as determined by Duncan’s Multiple Range Test (DMRT).

**Table 3:** Effect of seeding ratios and Italian ryegrass genotypes on dry matter accumulation from 2014 to 2018.

Treatment	2014–2015	2015–2016	2016–2017	2017–2018
**T_1_: Punjab ryegrass-1 + 100:0**	11.90^bc^	12.39^b^	12.98^c^	13.79^b^
**T_2_: Punjab ryegrass-1 + 75:25**	11.42^cd^	12.01^b^	12.31^d^	12.54^c^
**T_3_: Punjab ryegrass-1 + 50:50**	10.27^f^	10.95^d^	11.17^f^	11.41^d^
**T_4_: Punjab ryegrass-1 + 25:75**	8.68^gh^	9.22^ef^	9.59^hi^	9.81^e^
**T_5_: Kashmir Collection + 100:0**	10.75^ef^	11.13^cd^	11.68^ef^	12.09^c^
**T_6_: Kashmir Collection + 75:25**	9.29^g^	9.80^e^	10.29^g^	10.83^d^
**T_7_: Kashmir Collection + 50:50**	8.30^h^	9.05^f^	9.26^i^	9.70^e^
**T_8_: Kashmir Collection + 25:75**	7.17^i^	7.95^g^	8.12^j^	8.53^f^
**T_9_: *Makhan* Grass + 100:0**	13.20^a^	13.72^a^	14.17^a^	14.73^a^
**T_10_: *Makhan* Grass + 75:25**	12.47^b^	13.09^a^	13.57^b^	14.41^a^
**T_11_: *Makhan* Grass + 50:50**	11.03^de^	11.72^bc^	12.09^de^	12.30^c^
**T_12_: *Makhan* Grass + 25:75**	9.09^g^	9.75^e^	9.90^gh^	9.95^e^
**T_13_: Sole Egyptian clover**	6.44^j^	7.00^h^	7.53^k^	7.99^f^
**SEM±**	0.160	0.188	0.158	0.175
**LSD (*p*< 0.05)**	0.469	0.553	0.463	0.514

Treatment means with different lowercase letters are significant different as determined by Duncan’s Multiple Range Test (DMRT).

### 3.2 Crude protein concentration and yield

The analysis of crude protein concentration revealed a spectrum spanning from 11.37% to 19.83%. A markedly elevated crude protein concentration of 19.83% was notably discerned in the sole stand of Egyptian clover. The sole stand of Egyptian clover performed similarly across the experimental years from 2014 to 2018 in terms of crude protein concentration (**Table 4**). Concurrently, combinations featuring Egyptian clover in higher proportions, specifically in the seeding ratios of Punjab ryegrass-1 (25:75) and *Makhan* grass (25:75), exhibited heightened crude protein concentration, reaching 18.08% subsequent to the sole stand of Egyptian clover. Conversely, the lowest crude protein concentration was noted in the sole stand of Kashmir collection at 11.37%, a figure statistically at par with the sole stand of the Punjab ryegrass-1 (11.66%) genotype of Italian ryegrass. The performance of the sole stand of the Kashmir collection genotype was consistently poor for all the experimental years, i.e., from 2014 to 2018 (**Table 5**). Although the crude protein concentration was the highest in the sole cultivation of Egyptian clover, a significantly elevated crude protein yield of 1.92 Mg/ha was evident in the combination of *Makhan* grass and Egyptian clover at a seeding ratio of 50:50. This combination performed consistently better across the experimental years (2014–2018). This outcome was statistically at par with the same combination at seeding ratios of 75:25 (1.91 Mg/ha) and 25:75 (1.75 Mg/ha) (**Table 6**). Conversely, the combination of Kashmir collection and Egyptian clover at a seeding ratio of 75:25 yielded the lowest crude protein of 1.26 Mg/ha. This outcome was statistically at par with the sole cultivation of the Kashmir collection, which exhibited a crude protein yield of 1.29 Mg/ha.

**Table 4:** Effect of seeding ratios and Italian ryegrass genotypes on crude protein concentration from 2014 to 2018.

Crude protein concentration (%)				
Treatment	2014–2015	2015–2016	2016–2017	2017–2018
**T_1_: Punjab ryegrass-1 + 100:0**	12.1^g^	11.9^h^	11.5^h^	11.2^h^
**T_2_: Punjab ryegrass-1 + 75:25**	13.8^ef^	13.4^fg^	13.3^fg^	13.2^fg^
**T_3_: Punjab ryegrass-1 + 50:50**	15.8^cd^	15.3^de^	15.1^de^	15.0^de^
**T_4_: Punjab ryegrass-1 + 25:75**	18.5^b^	18.1^b^	17.9^b^	17.7^b^
**T_5_: Kashmir Collection + 100:0**	11.8^g^	11.5^h^	11.2^h^	11.0^h^
**T_6_: Kashmir Collection + 75:25**	12.9^fg^	12.6^gh^	12.4^gh^	12.2^gh^
**T_7_: Kashmir Collection + 50:50**	15.3^d^	14.9^e^	14.8^e^	14.5^e^
**T_8_: Kashmir Collection + 25:75**	17.1^c^	16.7^c^	16.5^c^	16.3^c^
**T_9_: *Makhan Grass* + 100:0**	13.9^ef^	13.5^fg^	13.3^fg^	13.0^fg^
**T_10_: *Makhan Grass* + 75:25**	14.7^de^	14.4^ef^	14.1^ef^	13.9^ef^
**T_11_: *Makhan Grass* + 50:50**	16.7^c^	16.4^cd^	16.2^cd^	16.0^cd^
**T_12_: *Makhan Grass* + 25:75**	18.4^b^	18.1^b^	17.9^b^	17.9^b^
**T_13_: Sole Egyptian clover**	20.3^a^	19.9^a^	19.7^a^	19.4^a^
**SEM±**	0.274	0.280	0.266	0.259
**LSD (*p*< 0.05)**	0.804	0.823	0.780	0.760

Treatment means with different lowercase letters are significant different as determined by Duncan’s Multiple Range Test (DMRT).

**Table 5:** Effect of seeding ratios and Italian ryegrass genotypes on crude protein yield from 2014 to 2018.

Crude protein yield (Mg/ha)				
Treatment	2014–2015	2015–2016	2016–2017	2017–2018
**T_1_: Punjab ryegrass-1 + 100:0**	1.44^c^	1.47^e^	1.50^e^	1.54^d^
**T_2_: Punjab ryegrass-1 + 75:25**	1.57^b^	1.61^d^	1.63^d^	1.65^cd^
**T_3_: Punjab ryegrass-1 + 50:50**	1.62^b^	1.68^cd^	1.69^cd^	1.71^bc^
**T_4_: Punjab ryegrass-1 + 25:75**	1.61^b^	1.67^cd^	1.72^cd^	1.74^bc^
**T_5_: Kashmir Collection + 100:0**	1.26^d^	1.28^fg^	1.31^f^	1.33^e^
**T_6_: Kashmir Collection + 75:25**	1.19^d^	1.24^g^	1.28^f^	1.32^e^
**T_7_: Kashmir Collection + 50:50**	1.27^d^	1.35^efg^	1.37^ef^	1.41^e^
**T_8_: Kashmir Collection + 25:75**	1.22^d^	1.32^fg^	1.34^f^	1.39^e^
**T_9_: *Makhan Grass* + 100:0**	1.84^a^	1.85^ab^	1.88^ab^	1.92^a^
**T_10_: *Makhan Grass* + 75:25**	1.83^a^	1.89^ab^	1.92^a^	2.00^a^
**T_11_: *Makhan Grass* + 50:50**	1.84^a^	1.92^a^	1.96^a^	1.97^a^
**T_12_: *Makhan Grass* + 25:75**	1.67^b^	1.76^bc^	1.77^bc^	1.78^b^
**T_13_: Sole Egyptian clover**	1.31^cd^	1.40^ef^	1.48^e^	1.55^d^
**SEM±**	0.037	0.037	0.035	0.033
**LSD (*p*< 0.05)**	0.107	0.109	0.102	0.097

Treatment means with different lowercase letters are significant different as determined by Duncan’s Multiple Range Test (DMRT).

**Table 6:** Effect of seeding ratios and Italian ryegrass genotypes on total green forage harvested, dry matter accumulated, crude protein concentration, and yield.

Treatment	Green forage harvested (Mg/ha)	Dry matter accumulated (Mg/ha)	Crude protein concentration (%)	Crude protein yield (Mg/ha)
**T_1_: Punjab ryegrass-1 + 100:0**	59.69^bc^	12.76^bc^	11.66^h^	1.49^def^
**T_2_: Punjab ryegrass-1 + 75:25**	61.23^bc^	12.06^cd^	13.41^fg^	1.62^cde^
**T_3_: Punjab ryegrass-1 + 50:50**	57.18^c^	10.95^e^	15.29^cde^	1.67^cd^
**T_4_: Punjab ryegrass-1 + 25:75**	49.70^d^	9.32^fg^	18.08^b^	1.68^bc^
**T_5_: Kashmir Collection + 100:0**	49.97^d^	11.41^de^	11.37^h^	1.29^g^
**T_6_: Kashmir Collection + 75:25**	45.47^e^	10.05^f^	12.54^gh^	1.26^g^
**T_7_: Kashmir Collection + 50:50**	42.38^ef^	9.07^g^	14.87^def^	1.35^fg^
**T_8_: Kashmir Collection + 25:75**	41.46^ef^	7.94^h^	16.62^bc^	1.32^fg^
**T_9_: *Makhan* Grass + 100:0**	65.61^a^	13.95^a^	13.41^fg^	1.87^ab^
**T_10_: *Makhan* Grass + 75:25**	62.71^ab^	13.38^ab^	14.29^ef^	1.91^a^
**T_11_: *Makhan* Grass + 50:50**	61.77^ab^	11.78^de^	16.33^cd^	1.92^a^
**T_12_: *Makhan* Grass + 25:75**	51.89^d^	9.67^fg^	18.08^b^	1.75^abc^
**T_13_: Sole Egyptian clover**	40.96^f^	7.24^h^	19.83^a^	1.44^efg^
**SEM±**	0.742	0.153	0.264	0.03
**LSD (*p*< 0.05)**	2.179	0.448	0.776	0.10

Treatment means with different lowercase letters are significant different as determined by Duncan’s Multiple Range Test (DMRT).

### 3.3 Effect of seeding ratios and Italian ryegrass genotypes on soil organic carbon and available nitrogen

The exclusive cultivation of either Egyptian clover or Italian ryegrass genotypes, as well as their various combinations across diverse seeding ratios, exhibited no discernible impact on soil organic carbon levels. Conversely, the presence of distinct treatment combinations exerted a pronounced influence on soil available nitrogen content, with values ranging from 218.25 to 246.75 kg/ha. Notably, a statistically significant increase in available nitrogen content, reaching 246.75 kg/ha, was observed in the sole stand of Egyptian clover. This finding was at par with comparable levels achieved in the combinations featuring higher proportions of Egyptian clover, namely, Punjab ryegrass-1 (25:75), Kashmir collection (25:75), and *Makhan* grass (25:75), recording available nitrogen levels of 242.25, 232.58, and 237.83 kg/ha, respectively. Conversely, the lowest available nitrogen content of 218.25 kg/ha manifested in the sole stand of the Italian ryegrass genotype, i.e., Punjab ryegrass-1. This result demonstrated statistical parity with the exclusive stands of the Kashmir collection and *Makhan* grass (**Table 7**).

**Table 7:** Effect of seeding ratios and Italian ryegrass genotypes on soil organic carbon, available nitrogen, net returns, and benefit–cost ratio.

Treatment	Soil organic carbon (kg/ha)	Available nitrogen (kg/ha)	Net returns (₹/ha)	Benefit–cost ratio
**T_1_: Punjab ryegrass-1 + 100:0**	6.86	218.25^d^	1059.62^ab^	2.17^a^
**T_2_: Punjab ryegrass-1 + 75:25**	6.71	229.33^bcd^	968.49^c^	1.82^bc^
**T_3_: Punjab ryegrass-1 + 50:50**	6.97	238.33^abc^	998.02^c^	1.93^bc^
**T_4_: Punjab ryegrass-1 + 25:75**	6.63	242.25^ab^	877.95^d^	1.77^cd^
**T_5_: Kashmir Collection + 100:0**	6.96	218.50^d^	831.26^d^	1.77^de^
**T_6_: Kashmir Collection + 75:25**	6.76	221.00^d^	724.30^e^	1.58^de^
**T_7_: Kashmir Collection + 50:50**	6.75	226.58^cd^	710.69^ef^	1.58^de^
**T_8_: Kashmir Collection + 25:75**	6.82	232.58^abcd^	653.96f	1.46^e^
**T_9_: *Makhan* Grass + 100:0**	6.86	226.75^cd^	1085.65^a^	2.01^ab^
**T_10_: *Makhan* Grass + 75:25**	6.77	227.75^bcd^	1071.92^ab^	2.01^ab^
**T_11_: *Makhan* Grass + 50:50**	6.82	230.33^bcd^	1016.10^bc^	1.90^bc^
**T_12_: *Makhan* Grass + 25:75**	6.98	237.83^abc^	880.74^d^	1.77^cd^
**T_13_: Sole Egyptian clover**	6.77	246.75^a^	538.54^g^	1.19^f^
**SEM±**	0.13	2.76	15.891	0.04
**LSD (*p*< 0.05)**	NS	8.12	46.658	0.12

Treatment means with different lowercase letters are significant different as determined by Duncan’s Multiple Range Test (DMRT). NS, Non-significant.

### 3.4 Effect of seeding ratios and Italian ryegrass genotypes on economics

Net returns exhibited a range from 538.54 USD/ha to 1,085.65 USD/ha, while the benefit–cost ratios spanned from 1.19 to 2.17. These variations were observed in response to the combinations of Egyptian clover and genotypes of Italian ryegrass cultivated under diverse seeding ratios, as detailed in **Table 7**. The highest net returns, amounting to 1,085.65 USD/ha, were achieved through the sole cultivation of the Italian ryegrass genotype, *Makhan* grass. This performance was statistically comparable to the sole cultivation of Punjab ryegrass-1 (1059.62 USD) and the co-cultivation of *Makhan* grass and Egyptian clover at seeding ratios of 75:25 (1071.92 USD/ha). Assessing the benefit–cost ratio, the sole cultivation of the Punjab ryegrass-1 genotype emerged as the most economically efficient, attaining a ratio of 2.17. This performance was statistically at par with the sole stand of *Makhan* grass (2.01) and the combination of *Makhan* grass with Egyptian clover at a seeding ratio of 75:25 (2.01). Conversely, the exclusive cultivation of Egyptian clover yielded the lowest net returns of 538.54 USD/ha, accompanied by a benefit–cost ratio of 1.19.

### 3.5 Effect of seeding ratios and Italian ryegrass genotypes on competition indices

#### 3.5.1 Land equivalent ratio

**Figure 1:**
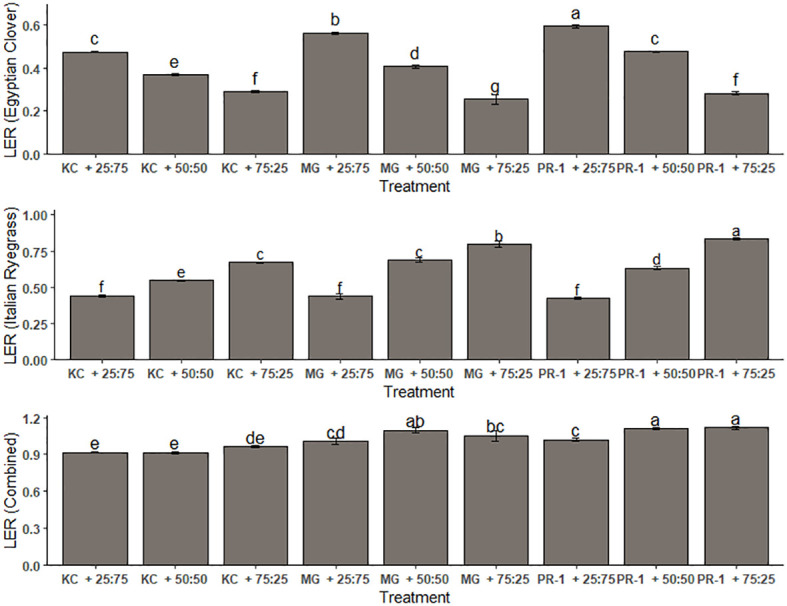
Effect of seeding ratios and Italian ryegrass genotypes on land equivalent ratio (LER). Different lowercase letters define significant differences among treatment means as determined by Duncan’s Multiple Range Test (DMRT).

The value of LER for Italian ryegrass ranged from 0.43 to 0.83 and that for Egyptian clover spanned from 0.25 to 0.59, whereas the total LER ranged from 0.92 to 1.11 (**Figure 1**). The highest LER in terms of Egyptian clover was evaluated with the co-cultivation of Punjab ryegrass-1 and Egyptian clover with a seeding ratio of 25:75 (0.59), and the lowest LER was evaluated with the combination of *Makhan* grass and Egyptian clover with a seeding ratio of 75:25 (0.25). The corresponding values in terms of Italian ryegrass were obtained with the co-cultivation of Punjab ryegrass-1 and Egyptian clover with seed ratios of 75:25 (0.83) and 25:75 (0.43). The highest total LER of 1.11 was with the combination of Punjab ryegrass-1 and Egyptian clover with seeding ratios of 75:25 and 50:50, whereas the lowest total LER of 0.92 was with the combination of Kashmir collection and Egyptian clover at a seed ratio of 50:50.

#### 3.5.2 Competitive ratio

**Figure 2:**
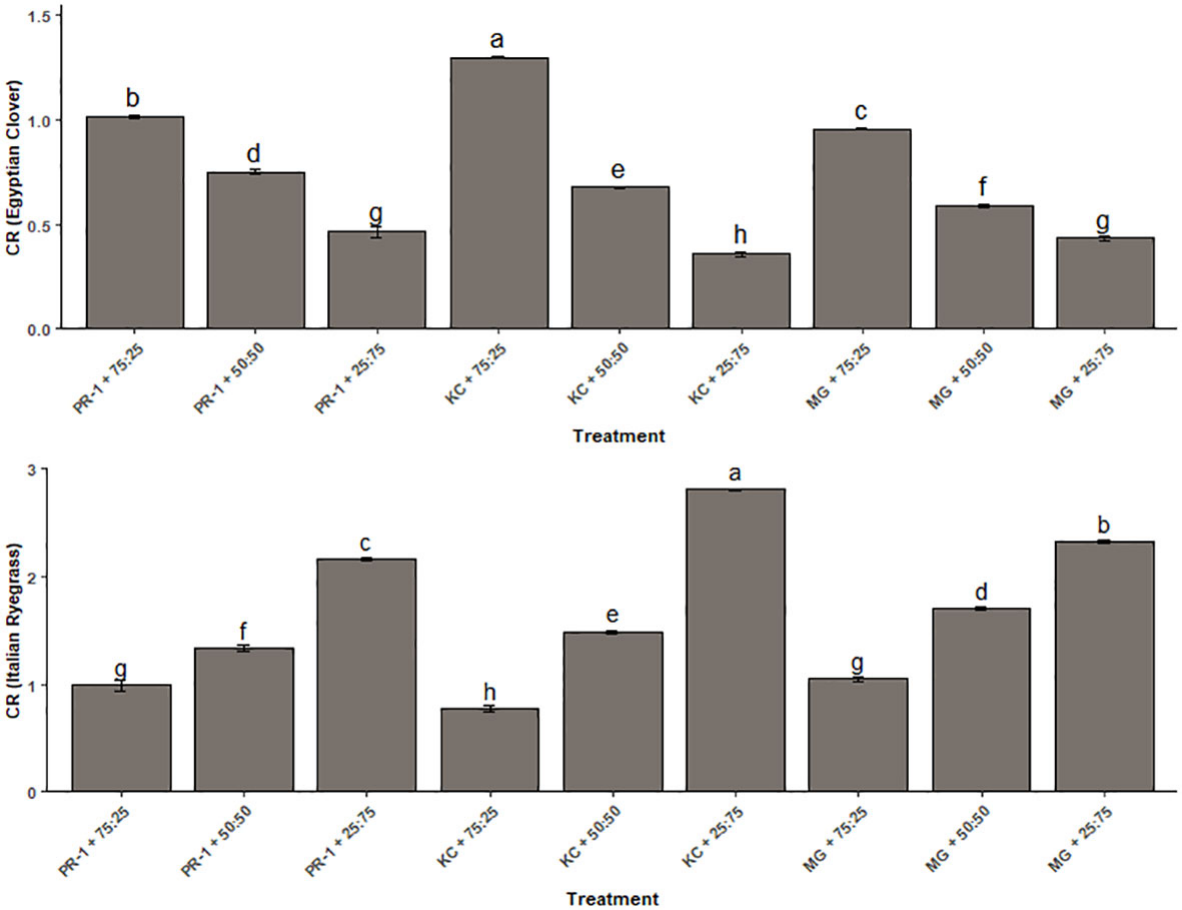
Effect of seeding ratios and Italian ryegrass genotypes on crop competitive ratios. Different lowercase letters define significant differences among treatment means as determined by Duncan’s Multiple Range Test (DMRT).

The combination of seeding ratios and Italian ryegrass genotypes influenced Egyptian clover CR values significantly, which ranged from 0.43 to 1.30, while for Italian ryegrass, it spanned between 0.77 and 2.80 (**Figure 2**). The range itself presented high competitiveness of Italian ryegrass in comparison to its companion crop Egyptian clover. The highest CR of 2.80 with respect to Italian ryegrass was observed with the Kashmir collection in combination with Egyptian clover having a seeding ratio of 25:75, while with respect to Egyptian clover, the highest CR of 1.30 was with the combination of the Kashmir collection and Egyptian clover but with a seeding ratio of 75:25. The lowest CR of 0.43 with respect to Egyptian clover was evaluated with the co-cultivation of *Makhan* grass and Egyptian clover with a seeding ratio of 25:75, whereas the lowest CR of 0.77 in terms of Italian ryegrass was evaluated with the co-cultivation of the Kashmir collection with a seed ratio of 75:25.

#### 3.5.3 Relative crowding coefficient

**Figure 3:**
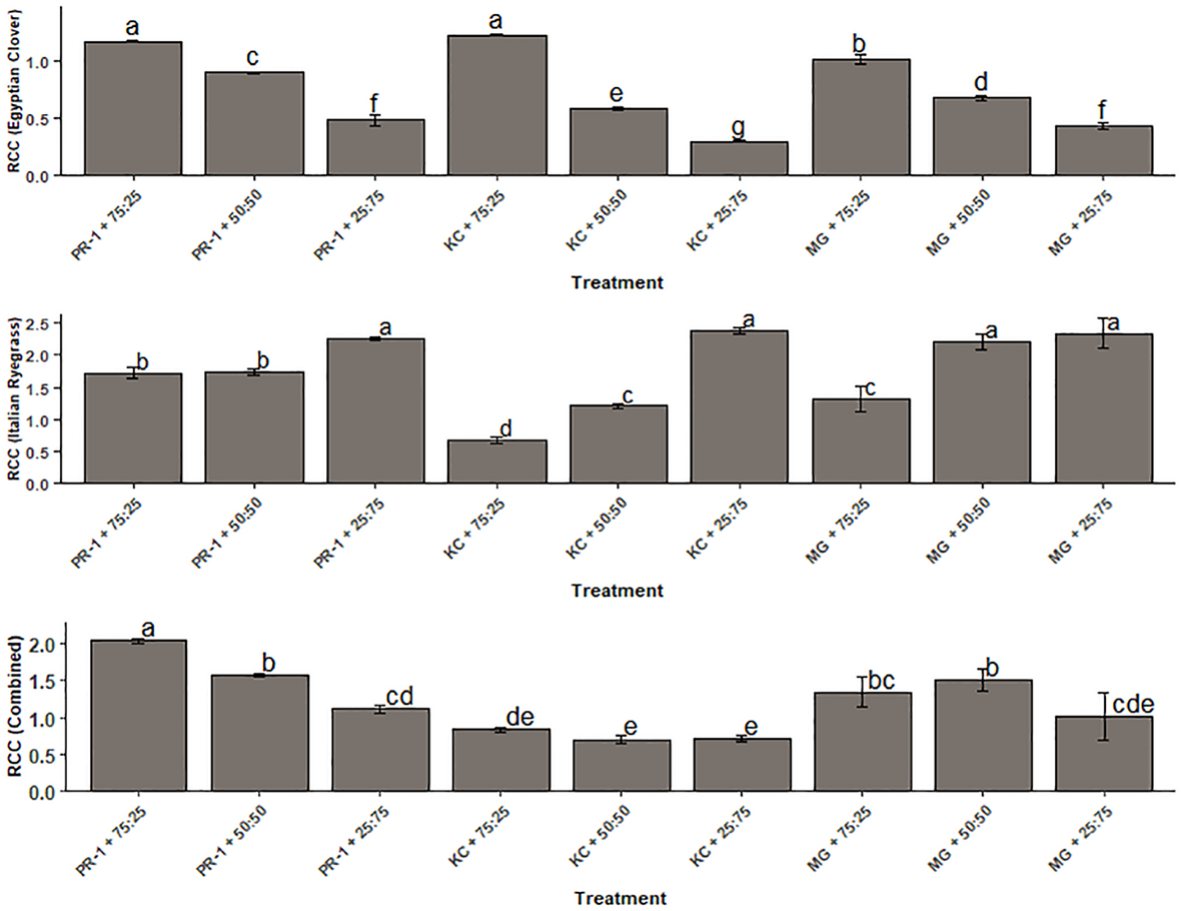
Effect of seeding ratios and Italian ryegrass genotypes on relative crowding coefficient (*K*). Different lowercase letters define significant differences among treatment means as determined by Duncan’s Multiple Range Test (DMRT).

The value of *K* for Italian ryegrass ranged from 0.68 to 2.38; for Egyptian clover, it spanned from 0.30 to 1.23, whereas the total K ranged from 0.70 to 2.04 (**Figure 3**). The highest and lowest *K* in terms of Egyptian clover were evaluated with the co-cultivation of the Kashmir collection and Egyptian clover with seeding ratios of 75:25 (1.23) and 25:75 (0.30), respectively. The corresponding values in terms of Italian ryegrass were obtained with the co-cultivation of the Kashmir collection and Egyptian clover with seed ratios of 25:75 (2.38) and 75:25 (0.68). The highest total *K* of 2.04 was with the combination of Punjab ryegrass-1 and Egyptian clover with a seeding ratio of 75:25, whereas the lowest total K of 0.70 was with the combination of the Kashmir collection and Egyptian clover at a seed ratio of 50:50.

#### 3.5.4 Actual yield loss

**Figure 4:**
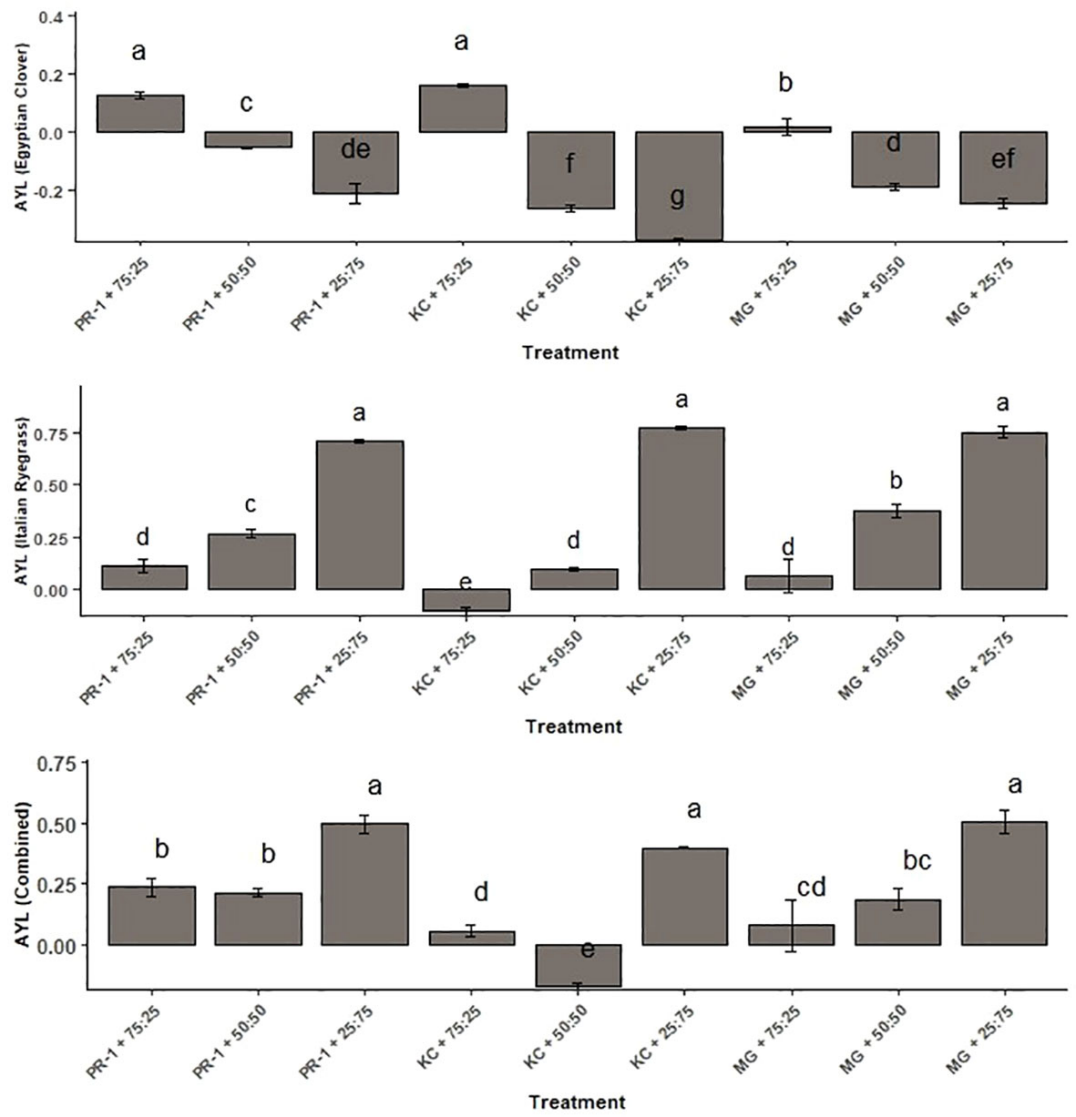
Effect of seeding ratios and Italian ryegrass genotypes on the actual yield loss (AYL). Different lowercase letters define significant differences among treatment means as determined by Duncan’s Multiple Range Test (DMRT).

The value of AYL for Italian ryegrass ranged from −0.10 to 0.77; for Egyptian clover, it spanned from −0.37 to 0.16, whereas the total AYL ranged from −0.17 to 0.50 (**Figure 4**). The highest and lowest AYL in terms of Egyptian clover were evaluated with the co-cultivation of the Kashmir collection and Egyptian clover with seeding ratios of 75:25 (0.16) and 25:75 (−0.37), respectively. The corresponding values in terms of Italian ryegrass were obtained with the co-cultivation of Kashmir collection and Egyptian clover with seed ratios of 25:75 (0.77) and 75:25 (−0.10). The highest total AYL of 0.50 was with the combination of *Makhan* grass with Egyptian clover with a seeding ratio of 25:75, whereas the lowest total AYL of −0.17 was with the combination of the Kashmir collection and Egyptian clover at a seed ratio of 50:50.

### 3.6 Correlation analysis between harvest, quality parameters, and competitive indices

**Figure 5:**
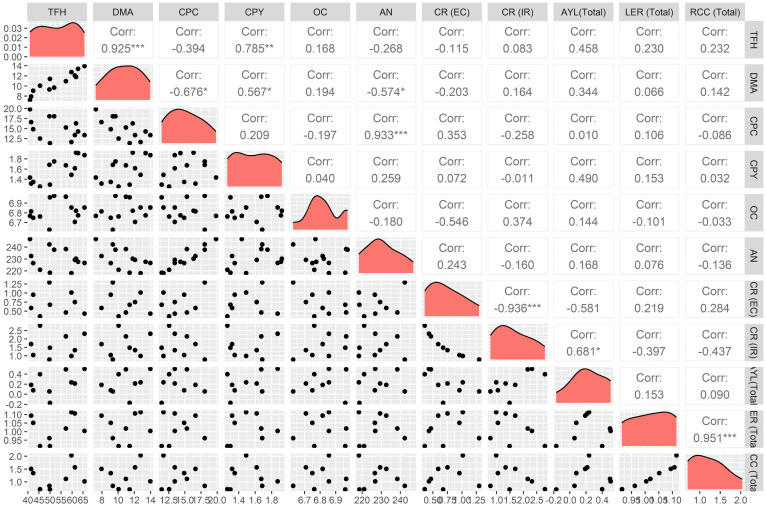
Correlation studies among TFH, DMA, CPC, CPY, competitive indices, and soil properties. DMA, Dry Matter Accumulation (Mg/ha); TFH, Total Forage Harvest (Mg/ha); CPY, Crude Protein Yield (Mg/ha); RCC (Combined), relative crowing coefficient (combined); LER, Land Equivalent Ratio (Combined); AYL (Combined), Actual Yield Loss (Combined); CR (IR), competitive ratio (Italian ryegrass); OC, organic carbon (kg/ha); AN, Available Nitrogen (kg/ha); CPC, Crude Protein Concentration (%); and CR (EC), Competitive ratio (Egyptian Clover). *, **, and *** represent significance at *p*-values of 0.05, 0.01, and 0.001, respectively.

The results for the correlation are represented in **Figure 5**. The forage harvest parameters were found to be positively correlated among each other. This suggests that an increase in one of the parameters would result in an increase of the other. Total forage harvested (TFH) was found to be strongly correlated to dry matter yield (DMY) (*r* = 0.925) and crude protein yield (CPY) (*r* = 0.785), whereas moderate correlation between dry matter accumulation and crude protein yield was observed (*r* = 0.567). In regard to the competitive indices, moderate correlation was observed between TFH and AYL (total) (*r* = 0.458) as well as for CPY and AYL (combined) (*r* = 0.490). As expected, CPC showed a negative correlation with DMA (*r* = −0.676) but a positive correlation with available soil nitrogen (0.933). The above results clearly state a scenario of decreasing CPC with increase in DMA in cropping mixtures. Available nitrogen with a positive relationship with CPC states that with the increase in soil available nitrogen, plants will surely have an increased CPC in their dry matter. A moderate but a negative correlation was observed within AN and DMA (*r* = 0.574).

## 4 Discussion

The forage grasses have been known to have considerably higher forage producing capacity. The potential of Italian ryegrass to produce significantly higher dry matter or forage harvest could be attributed to its robust photosynthetic capacity (Helmy et al., 2011). A notable decline in the forage harvested is observed when the seeding fraction of Italian ryegrass is reduced from 100 to 25. This discernible reduction in forage harvest may be attributed to the inherent higher harvest potential of grasses in contrast to leguminous counterparts (Bacchi et al., 2021). A field investigation by Bacchi et al. (2021) reported similar outcomes wherein grasses outperformed legumes in terms of total forage harvest and dry matter accumulation. Similarly, Prajapati and his co-workers in 2020 (Prajapati et al., 2020) reported that the sole ryegrass stand had a considerably higher forage harvest as compared to the sole stand of Egyptian clover. Furthermore, it was reiterated that intercropping systems of Italian ryegrass and Egyptian clover outperform monocultures of Egyptian culture in terms of forage harvest and dry matter accumulation. Monocultures of Italian ryegrass performed at par with the cropping mixtures of Italian ryegrass and clover (Prajapati et al., 2020). The variation in dry matter accumulated can be ascribed to the seeding proportions of Italian ryegrass and Egyptian clover, and green forage harvest for the various combinations. Higher green forage harvest with the sole stand of Italian ryegrass resulted in higher dry matter accumulation. The findings are in conformity with the research conducted by Yucel et al. (2018).

The capacity to fix atmospheric nitrogen by leguminous plants augments the protein concentration within the plants (Salama, 2020). In the present investigation, increase in the seeding proportion of leguminous Egyptian clover while co-cultivating with Italian ryegrass enhanced the crude protein concentration, which aligns with the findings of Prajapati et al. (2020). The pragmatic approach to compensate for high-quality forage would be to mix leguminous crops and grasses in right proportions. Crude protein yield is a function of dry matter accumulated and crude protein concentration; hence, low dry matter accumulation would result in decline in crude protein yield. The profitability of the various treatments was proportional to the green forage production of the forage combination. The sole stand of Italian ryegrass, i.e., Punjab ryegrass-1, had a better benefit–cost ratio and net returns. As discussed previously, the nitrogen-fixing ability inherent in leguminous crops, i.e., Egyptian clover, extends beyond its role in elevating crude protein concentration within forage blends. Significantly, this biological process contributes to the improvement of nitrogen availability in the soil, thereby nurturing a conducive environment for enhanced agricultural productivity. This dual functionality, encompassing both nutritional improvement in forage crops and soil nitrogen enhancement, emphasizes the multifaceted contributions of leguminous crops to sustainable agronomic practices.

The CR for Italian ryegrass was found to be considerably greater than that of its component crop, Egyptian clover, indicating that Italian ryegrass is a more competitive crop in forage combinations (Dhima et al., 2007). In the present investigation, intraspecific rivalry among crops had a stronger impact on competitive abilities. Increased seeding proportion led to increased intraspecific competition for both Italian ryegrass and Egyptian clover, negatively affecting their CR. With a drop in Italian ryegrass sowing proportion or a rise in legume crop sowing ratio, Egyptian clover CR declined whereas lowering the fraction of Egyptian clover sown had a favorable effect on CR (Egyptian clover). *Makhan* grass’s stronger producing capacity and aggressive growth habit resulted in much higher CR (rye) values for ryegrass and lower CR values for Egyptian clover. With its slower growth, the Kashmir collection allowed Egyptian clover to flourish at a considerable rate and compete with Italian ryegrass.

Except at 25:75 seeding ratio, Italian ryegrass showed partial LER values larger than 0.5 and yield advantage at different sowing ratios and genotypes. Total land equivalent ratios greater than 1 indicated that the forage blends had a yield advantage. Such LER values demonstrated the ability of grass–legume mixes to provide greater yield than their mono-cropping counterparts (Bedoussac and Justes, 2010; Vlachostergios et al., 2018). It has been found that grass–legume combinations may efficiently use environmental and land resources (Dhima et al., 2014). In comparison to Punjab Ryegrass-1 and 75:25 seeding proportion-based co-cultivation, sole cropping would have required 1.114 times more land area to yield the same amount of forage harvest, whereas sole cropping with a Punjab Ryegrass-based 50:50 seeding ratio would require 1.107 times more space to produce the same amount of harvest. Among genotypes, *Makhan* grass combination with Egyptian clover performed equivalent to the co-cultivated Punjab ryegrass-1 and Egyptian clover but much better than the sole stand of Egyptian clover in terms of resource use. *K* (ryegrass) was found to be considerably higher than *K* (Egyptian clover), indicating that Italian ryegrass is a more competitive crop than Egyptian clover. Most of the *K* (Egyptian clover) values under the impact of combinations of genotypes and seeding proportions were less than 1, suggesting that the relevant treatments had a yield disadvantage (Willey and Rao, 1980; Ghosh, 2004). Except for the Kashmir collection genotype with 75:25 planting percentage, *K* (ryegrass) values were typically more than 1, indicating a yield advantage (Banik et al., 2000).

Partial AYL values for Italian ryegrass demonstrated a clear yield benefit for the crop when Egyptian clover was included (Rady, 2016). As the seeding ratio of Egyptian clover was increased, the yield advantage of Italian ryegrass rose. This was mostly due to Egyptian clover’s capacity to fix atmospheric nitrogen, which may be efficiently absorbed by partner cereals/grasses and supplied to soil following the breakdown of legume biomass. Italian ryegrass was also found to have yield advantages over Egyptian clover. Banik et al. (2000) also observed that Italian ryegrass was extending yield advantages to Egyptian clover. However, the yield advantage was continually decreasing as the fraction of Egyptian clover sowing rose, mostly due to intraspecific competition with Egyptian clover plants. Among genotypes, *Makhan* grass’s better yielding potential provided much bigger yield benefits to Italian ryegrass and significantly lesser advantages to Egyptian clover.

## 5 Conclusion

With their great photosynthetic efficiency, grasses contribute significantly to the world livestock’s feed supply. However, giving animals low-quality feed, particularly in terms of protein concentration, has a detrimental influence on livestock output. Forage combinations based on grass and legumes can help to overcome the problem of low-quality feed. Grass genotypes and grass–legume-based seeding ratios, with widely varied dry matter contributions, have a substantial impact on the productivity, profitability, and nutritive value of forage combinations. Experiment results with Italian ryegrass genotypes and Italian ryegrass: Egyptian clover seeding ratios over the years revealed that mixed planting of Italian ryegrass and Egyptian clover was the best option for supplying excellent forage. Although sole stands of the Italian ryegrass genotype, i.e., *Makhan* grass, led to 60% more forage harvests compared to the sole stand of Egyptian clover followed by cropping mixtures with Egyptian clover at seeding ratios of 75:25 (53% yield increment) and 50:50 (51% yield increment), the nutritive value in terms of crude protein yield was considerably higher for 50:50 and 75:25 seeding ratios of Italian ryegrass and Egyptian clover, i.e., approximately 33% and 30% higher crude protein yield compared to the sole stand of Egyptian clover. Owing to substantially higher forage harvests, Italian ryegrass alone proved far more lucrative than mixed forage stands of Italian ryegrass and Egyptian clover. Over time, an increased amount of Egyptian clover had a favorable impact on soil fertility. *Makhan* grass outperformed other genotypes and proved to be the most productive and profitable among them. In terms of forage output and revenue generation, the optimum seeding ratio and genotype combination was *Makhan* grass with a 100:0 seeding ratio with almost double the net returns as compared to returns generated from the sole stand of Egyptian clover. Thus, the 100:0 planting ratio with *Makhan* grass is the best choice for increased productivity and profitability, although a 75:25 seeding ratio with *Makhan* grass might be a reasonable alternative in terms of quality feed and soil fertility. According to competition indices such as CR and relative crowding coefficient, Italian ryegrass was a moderately competitive crop and Egyptian clover was a relatively dominating crop. AYL values confirmed the complementary relationship between grasses and legumes, with Egyptian clover having significantly higher AYL values and yield advantage at 75:25 seeding proportion, whereas Italian ryegrass had it at 25:75 seeding proportion, i.e., with higher legume inclusion.

## Data availability statement

The original contributions presented in the study are included in the article/**Supplementary Material**. Further inquiries can be directed to the corresponding author.

## Author contributions

NK: Conceptualization, Methodology, Project administration, Writing – review & editing. RS: Conceptualization, Investigation, Methodology, Writing – review & editing. RA: Conceptualization, Methodology, Project administration, Writing – review & editing. GS: Conceptualization, Methodology, Project administration, Writing – review & editing. AS: Formal analysis, Writing – review & editing. TS: Formal analysis, Writing – original draft. RR: Funding acquisition, Writing – review & editing.
