# Supplementary material for: Optimizing forage harvest and the nutritive value of Italian ryegrass-based mixed forage cropping under northwestern Himalayan conditions
Source: Front Plant Sci. 2024 Jul 3;15:1346936. doi: 10.3389/fpls.2024.1346936 (PMC11255485; doi:10.3389/fpls.2024.1346936)
Supplement: Supplementary file 1 [file Table_1.docx]

**Effect of seeding ratios and Italian ryegrass genotypes on actual yield loss of Egyptian clover**

| **Treatment** | **2014-15** | **2015-16** | **2016-17** | **2017-18** |
| --- | --- | --- | --- | --- |
| **Punjab ryegrass-1 + 75:25** | 0.25^a^ | 0.20^a^ | 0.09^a^ | -0.02^a^ |
| **Punjab ryegrass-1 + 50:50** | 0.05^b^ | -0.02^b^ | -0.08^b^ | -0.14^ab^ |
| **Punjab ryegrass-1 + 25:75** | -0.17^c^ | -0.19^c^ | -0.22^c^ | -0.26^bcd^ |
| **Kashmir Collection + 75:25** | 0.36^a^ | 0.22^a^ | 0.08^a^ | -0.01^a^ |
| **Kashmir Collection + 50:50** | -0.18^c^ | -0.23^c^ | -0.28^c^ | -0.35^cd^ |
| **Kashmir Collection + 25:75** | -0.32^d^ | -0.35^d^ | -0.39^c^ | -0.41^d^ |
| ***Makhan* Grass + 75:25** | 0.28^a^ | 0.07^b^ | -0.02^b^ | -0.24^bc^ |
| ***Makhan* Grass + 50:50** | -0.05^bc^ | -0.16^c^ | -0.25^c^ | -0.28^bcd^ |
| ***Makhan* Grass + 25:75** | -0.18^c^ | -0.23^c^ | -0.27^c^ | -0.29^bcd^ |
