# Supplementary material for: Optimizing forage harvest and the nutritive value of Italian ryegrass-based mixed forage cropping under northwestern Himalayan conditions
Source: Front Plant Sci. 2024 Jul 3;15:1346936. doi: 10.3389/fpls.2024.1346936 (PMC11255485; doi:10.3389/fpls.2024.1346936)
Supplement: Supplementary file 2 [file Table_2.docx]

**Effect of seeding ratios and Italian ryegrass genotypes on actual yield loss of Italian ryegrass**

| **Treatment** | **2014-15** | **2015-16** | **2016-17** | **2017-18** |
| --- | --- | --- | --- | --- |
| **Punjab ryegrass-1 + 75:25** | 0.08^d^ | 0.09^d^ | 0.12^d^ | 0.15^c^ |
| **Punjab ryegrass-1 + 50:50** | 0.19^c^ | 0.23^c^ | 0.29^c^ | 0.36^b^ |
| **Punjab ryegrass-1 + 25:75** | 0.51^a^ | 0.63^a^ | 0.79^a^ | 0.89^a^ |
| **Kashmir Collection + 75:25** | -0.14^e^ | -0.12^e^ | -0.09^e^ | -0.06^d^ |
| **Kashmir Collection + 50:50** | 0.01^d^ | 0.07^d^ | 0.11^d^ | 0.18^c^ |
| **Kashmir Collection + 25:75** | 0.55^a^ | 0.70^a^ | 0.82^a^ | 0.99^a^ |
| ***Makhan* Grass + 75:25** | 0.04^d^ | 0.06^d^ | 0.07^d^ | 0.08^c^ |
| ***Makhan* Grass + 50:50** | 0.32^b^ | 0.36^b^ | 0.39^b^ | 0.43^b^ |
| ***Makhan* Grass + 25:75** | 0.58^a^ | 0.68^a^ | 0.79^a^ | 0.93^a^ |
