# Supplementary material for: Optimizing forage harvest and the nutritive value of Italian ryegrass-based mixed forage cropping under northwestern Himalayan conditions
Source: Front Plant Sci. 2024 Jul 3;15:1346936. doi: 10.3389/fpls.2024.1346936 (PMC11255485; doi:10.3389/fpls.2024.1346936)
Supplement: Supplementary file 3 [file Table_3.docx]

**Effect of seeding ratios and Italian ryegrass genotypes on competitive ratio of Egyptian clover**

| **Treatment** | **2014-15** | **2015-16** | **2016-17** | **2017-18** |
| --- | --- | --- | --- | --- |
| **Punjab ryegrass-1 + 75:25** | 1.17^b^ | 1.10^b^ | 0.98^b^ | 0.85^b^ |
| **Punjab ryegrass-1 + 50:50** | 0.89^c^ | 0.80^c^ | 0.71^c^ | 0.63^cd^ |
| **Punjab ryegrass-1 + 25:75** | 0.56^e^ | 0.50^ef^ | 0.44^e^ | 0.39^ef^ |
| **Kashmir Collection + 75:25** | 1.59^a^ | 1.39^a^ | 1.19^a^ | 1.06^a^ |
| **Kashmir Collection + 50:50** | 0.82^cd^ | 0.72^cd^ | 0.65^c^ | 0.55^d^ |
| **Kashmir Collection + 25:75** | 0.44^e^ | 0.38^f^ | 0.33^f^ | 0.29^f^ |
| ***Makhan* Grass + 75:25** | 1.23^b^ | 1.01^b^ | 0.91^b^ | 0.70^c^ |
| ***Makhan* Grass + 50:50** | 0.72^d^ | 0.62^de^ | 0.54^d^ | 0.51^de^ |
| ***Makhan* Grass + 25:75** | 0.51^e^ | 0.46^f^ | 0.41^ef^ | 0.37^f^ |
