# Supplementary material for: Optimizing forage harvest and the nutritive value of Italian ryegrass-based mixed forage cropping under northwestern Himalayan conditions
Source: Front Plant Sci. 2024 Jul 3;15:1346936. doi: 10.3389/fpls.2024.1346936 (PMC11255485; doi:10.3389/fpls.2024.1346936)
Supplement: Supplementary file 4 [file Table_4.docx]

**Effect of seeding ratios and Italian ryegrass genotypes on competitive ratio of Italian ryegrass**

| **Treatment** | **2014-15** | **2015-16** | **2016-17** | **2017-18** |
| --- | --- | --- | --- | --- |
| **Punjab ryegrass-1 + 75:25** | 0.87^e^ | 0.92^ef^ | 1.03^e^ | 1.18^fg^ |
| **Punjab ryegrass-1 + 50:50** | 1.14^d^ | 1.26^d^ | 1.41^d^ | 1.61^de^ |
| **Punjab ryegrass-1 + 25:75** | 1.81^b^ | 2.03^b^ | 2.30^b^ | 2.56^b^ |
| **Kashmir Collection + 75:25** | 0.63^f^ | 0.72^f^ | 0.84^f^ | 0.96^g^ |
| **Kashmir Collection + 50:50** | 1.23^cd^ | 1.38^cd^ | 1.54^d^ | 1.82^cd^ |
| **Kashmir Collection + 25:75** | 2.29^a^ | 2.62^a^ | 2.99^a^ | 3.41^a^ |
| ***Makhan* Grass + 75:25** | 0.82^ef^ | 0.99^e^ | 1.09^e^ | 1.43^ef^ |
| ***Makhan* Grass + 50:50** | 1.39^c^ | 1.62^c^ | 1.86^c^ | 2.00^c^ |
| ***Makhan* Grass + 25:75** | 1.94^b^ | 2.17^b^ | 2.45^b^ | 2.72^b^ |
