# Supplementary material for: Optimizing forage harvest and the nutritive value of Italian ryegrass-based mixed forage cropping under northwestern Himalayan conditions
Source: Front Plant Sci. 2024 Jul 3;15:1346936. doi: 10.3389/fpls.2024.1346936 (PMC11255485; doi:10.3389/fpls.2024.1346936)
Supplement: Supplementary file 5 [file Table_5.docx]

**Effect of seeding ratios and Italian ryegrass genotypes on LER of Egyptian clover**

| **Treatment** | **2014-15** | **2015-16** | **2016-17** | **2017-18** |
| --- | --- | --- | --- | --- |
| **Punjab ryegrass-1 + 75:25** | 0.31^e^ | 0.30^d^ | 0.27^d^ | 0.24^e^ |
| **Punjab ryegrass-1 + 50:50** | 0.52^b^ | 0.49^b^ | 0.46^b^ | 0.43^bc^ |
| **Punjab ryegrass-1 + 25:75** | 0.62^a^ | 0.61^a^ | 0.58^a^ | 0.56^a^ |
| **Kashmir Collection + 75:25** | 0.34^e^ | 0.31^d^ | 0.27^d^ | 0.25^e^ |
| **Kashmir Collection + 50:50** | 0.41^d^ | 0.39^c^ | 0.36^c^ | 0.32^d^ |
| **Kashmir Collection + 25:75** | 0.51^bc^ | 0.49^b^ | 0.46^b^ | 0.44^b^ |
| ***Makhan* Grass + 75:25** | 0.32^e^ | 0.27^d^ | 0.24^d^ | 0.19^e^ |
| ***Makhan* Grass + 50:50** | 0.47^c^ | 0.42^c^ | 0.37^c^ | 0.36^cd^ |
| ***Makhan* Grass + 25:75** | 0.61^a^ | 0.58^a^ | 0.55^a^ | 0.54^a^ |
