# Supplementary material for: Optimizing forage harvest and the nutritive value of Italian ryegrass-based mixed forage cropping under northwestern Himalayan conditions
Source: Front Plant Sci. 2024 Jul 3;15:1346936. doi: 10.3389/fpls.2024.1346936 (PMC11255485; doi:10.3389/fpls.2024.1346936)
Supplement: Supplementary file 6 [file Table_6.docx]

**Effect of seeding ratios and Italian ryegrass genotypes on LER of Italian ryegrass**

| **Treatment** | **2014-15** | **2015-16** | **2016-17** | **2017-18** |
| --- | --- | --- | --- | --- |
| **Punjab ryegrass-1 + 75:25** | 0.81^a^ | 0.82^a^ | 0.84^a^ | 0.86^a^ |
| **Punjab ryegrass-1 + 50:50** | 0.59^c^ | 0.61^c^ | 0.64^d^ | 0.68^c^ |
| **Punjab ryegrass-1 + 25:75** | 0.38^e^ | 0.41^e^ | 0.45^f^ | 0.47^e^ |
| **Kashmir Collection + 75:25** | 0.64^b^ | 0.66^b^ | 0.68^c^ | 0.70^b^ |
| **Kashmir Collection + 50:50** | 0.50^d^ | 0.53^d^ | 0.55^e^ | 0.59^d^ |
| **Kashmir Collection + 25:75** | 0.39^e^ | 0.42^e^ | 0.46^f^ | 0.50^e^ |
| ***Makhan* Grass + 75:25** | 0.78^a^ | 0.79^a^ | 0.80^b^ | 0.81^a^ |
| ***Makhan* Grass + 50:50** | 0.66^b^ | 0.68^b^ | 0.70^c^ | 0.72^b^ |
| ***Makhan* Grass + 25:75** | 0.40^e^ | 0.42^e^ | 0.45^f^ | 0.48^e^ |
