# Supplementary material for: Optimizing forage harvest and the nutritive value of Italian ryegrass-based mixed forage cropping under northwestern Himalayan conditions
Source: Front Plant Sci. 2024 Jul 3;15:1346936. doi: 10.3389/fpls.2024.1346936 (PMC11255485; doi:10.3389/fpls.2024.1346936)
Supplement: Supplementary file 7 [file Table_7.docx]

**Effect of seeding ratios and Italian ryegrass genotypes on relative crowing coefficient of Egyptian clover**

| **Treatment** | **2014-15** | **2015-16** | **2016-17** | **2017-18** |
| --- | --- | --- | --- | --- |
| **Punjab ryegrass-1 + 75:25** | 1.37^a^ | 1.28^a^ | 1.13^a^ | 0.97^a^ |
| **Punjab ryegrass-1 + 50:50** | 1.11^b^ | 0.97^b^ | 0.84^c^ | 0.77^b^ |
| **Punjab ryegrass-1 + 25:75** | 0.56^de^ | 0.52^d^ | 0.47^ef^ | 0.42^cd^ |
| **Kashmir Collection + 75:25** | 1.54^a^ | 1.33^a^ | 1.11^a^ | 1.00^a^ |
| **Kashmir Collection + 50:50** | 0.70^cd^ | 0.63^cd^ | 0.56^de^ | 0.48^c^ |
| **Kashmir Collection + 25:75** | 0.35^e^ | 0.31^e^ | 0.28^g^ | 0.26^d^ |
| ***Makhan* Grass + 75:25** | 1.42^a^ | 1.10^b^ | 0.97^b^ | 0.70^b^ |
| ***Makhan* Grass + 50:50** | 0.90^c^ | 0.73^c^ | 0.60^d^ | 0.57^bc^ |
| ***Makhan* Grass + 25:75** | 0.53^de^ | 0.46^de^ | 0.41^f^ | 0.40^cd^ |
