# Supplementary material for: Optimizing forage harvest and the nutritive value of Italian ryegrass-based mixed forage cropping under northwestern Himalayan conditions
Source: Front Plant Sci. 2024 Jul 3;15:1346936. doi: 10.3389/fpls.2024.1346936 (PMC11255485; doi:10.3389/fpls.2024.1346936)
Supplement: Supplementary file 8 [file Table_8.docx]

**Effect of seeding ratios and Italian ryegrass genotypes on relative crowing coefficient of Italian ryegrass**

| **Treatment** | **2014-15** | **2015-16** | **2016-17** | **2017-18** |
| --- | --- | --- | --- | --- |
| **Punjab ryegrass-1 + 75:25** | 1.45^b^ | 1.59^b^ | 1.80^b^ | 2.15^b^ |
| **Punjab ryegrass-1 + 50:50** | 1.47^b^ | 1.61^b^ | 1.81^b^ | 2.12^b^ |
| **Punjab ryegrass-1 + 25:75** | 1.82^a^ | 2.08^a^ | 2.44^a^ | 2.71^a^ |
| **Kashmir Collection + 75:25** | 0.60^d^ | 0.65^d^ | 0.71^d^ | 0.80^d^ |
| **Kashmir Collection + 50:50** | 1.02^c^ | 1.14^c^ | 1.25^c^ | 1.45^c^ |
| **Kashmir Collection + 25:75** | 1.90^a^ | 2.21^a^ | 2.52^a^ | 2.97^a^ |
| ***Makhan* Grass + 75:25** | 1.19^bc^ | 1.29^bc^ | 1.35^c^ | 1.45^c^ |
| ***Makhan* Grass + 50:50** | 1.93^a^ | 2.12^a^ | 2.30^a^ | 2.52^ab^ |
| ***Makhan* Grass + 25:75** | 1.97^a^ | 2.16^a^ | 2.43^a^ | 2.82^a^ |
